# Osteoprotegerin autoantibodies do not predict low bone mineral density in middle-aged women

**DOI:** 10.1016/j.bonr.2017.10.004

**Published:** 2017-10-24

**Authors:** Fariba Vaziri-Sani, Charlotte Brundin, Daniel Agardh

**Affiliations:** a Kristianstad University, Kristianstad, Sweden; b Diabetes and Celiac Disease Unit, Department of Clinical Sciences, Lund University, Malmö, Sweden

**Keywords:** BMD, bone mineral density, DXA, dual-energy X-ray absorptiometry, DXA, OPG, osteprotegerin, RBA, radiobinding assays, Autoantibody, Bone mineral density, Osteopenia, Osteoporosis, Osteoprotegerin

## Abstract

**Purpose:**

Autoantibodies against osteoprotegerin (OPG) have been associated with osteoporosis. The aim was to develop an immunoassay for OPG autoantibodies and test their diagnostic usefulness of identifying women general population with low bone mineral density.

**Methods:**

Included were 698 women at mean age 55.1 years (range 50.4–60.6) randomly selected from the general population. Measurement of wrist bone mineral density (g/cm^2^) was performed of the non-dominant wrist by dual-energy X-ray absorptiometry (DXA). A T-score < − 2.5 was defined as having a low bone mineral density. Measurements of OPG autoantibodies were carried by radiobinding assays. Cut-off levels for a positive value were determined from the deviation from normality in the distribution of 398 healthy blood donors representing the 99.7th percentile.

**Results:**

Forty-five of the 698 (6.6%) women were IgG-OPG positive compared with 2 of 398 (0.5%) controls (*p* < 0.0001) and 35 of the 698 (5.0%) women had a T-score < − 2.5. There was no difference in bone mineral density between IgG-OPG positive (median 0.439 (range 0.315–0.547) g/cm^2^) women and IgG-OPG negative (median 0.435 (range 0.176–0.652) g/cm^2^) women (*p* = 0.3956). Furthermore, there was neither a correlation between IgG-OPG levels and bone mineral density (r_s_ = 0.1896; *p* = 0.2068) nor T-score (r_s_ = 0.1889; *p* = 0.2086). Diagnostic sensitivity and specificity of IgG-OPG for low bone mineral density were 5.7% and 92.9%, and positive and negative predictive values were 7.4% and 90.8%, respectively.

**Conclusion:**

Elevated OPG autoantibody levels do not predict low bone mineral density in middle-aged women selected from the general population.

## 1 Introduction

Osteoprotegerin (OPG) was discovered in 1997 as a novel protein involved in the regulation of bone density (Simonet et al., 1997). OPG belongs to the tumor necrosis factor receptor superfamily member 11B (TNFRSF11B), which acts as a cytokine receptor for receptor activator of nuclear factor kappa B ligand (RANKL), and is furthermore essential for the regulation of bone remodeling by maintaining the correct balance between bone resorption and bone formation (Delmas, 2008). In 2009, autoantibodies against OPG were discovered in a subset of patients with severe osteoporosis (Riches et al., 2009). Although, their role in the pathogenesis are still unclear, it has been suggested that OPG autoantibodies may neutralize OPG thereby leading to increased osteoclast activity causing bone resorption (Riches et al., 2009).

Radiobinding assays (RBA), or immunoprecipitation assays, measure antibodies bound to low amount of tracer radioactive antigen in a solution and antibodies directed against both linear and conformational epitopes are precipitated by conjugated beads (Grubin et al., 1994). By coupled in vitro transcription/translation of human plasmid cDNA, potentially any antigen in presence of radioactively labeled methionine can be used for the detection of autoantibodies and protocols can be further standardized for clinical practice. RBAs are both sensitive and specific for the detection of various autoantibodies related to human disease such as type 1 diabetes (Grubin et al., 1994) and celiac disease (Agardh et al., 2005), and moreover, proven suitable as a method of screening large populations (Bjorck et al., 2010). Still, there is paucity of RBAs for the assessment of OPG autoantibodies and no study has yet evaluated their usefulness as a method of screening the general population for osteoporosis.

The aims of this study were to develop an OPG autoantibody immunoassay and test the diagnostic usefulness of IgG-OPG to predict low bone mineral density (BMD) in middle-aged post-menopausal women selected from the general population. We hypothesized that IgG-OPG levels correlated with BMD and thus could be utilized as a potential screening marker of osteoporosis in the general population.

## 2 Material and methods

### 2.1 Subjects

A total of 10,766 women living in a defined area of Southern Sweden were identified through a population register and were asked to participate in the Women's Health in the Lund Area (WHILA) study between December 1, 1995 and February 3, 2000, and inclusion criteria and detailed characteristics are described elsewhere (Lidfeldt et al., 2002). Screening for osteoporosis was performed in 6917 post-menopausal women at mean age 56 years (range 50–64 years) from who a subset of blood samples were available from 698 women at mean age of 55.1 years (range 50.4–60.6) randomly selected for this study. As control subjects, we analyzed serum samples from 398 healthy blood donors (260 males, 133 females, 5 with unknown sex) at mean age 44.0 years of age. The Ethics committee at Malmö/Lund University approved the study (EPN 2011/9).

### 2.2 Bone mineral density (BMD) measurements

Measurement of wrist BMD was performed of the non-dominant wrist (at the 8 mm distal position) using dual-energy X-ray absorptiometry (DXT 200; Osteometer MediTech, Inc., Hawthorne, CA, USA) as previously described (Lidfeldt et al., 2002). In short, a phantom for daily calibration of the instrument was used, and one technician performed all measurements. BMD (g/cm^2^) was automatically compared with a “reference” population furnished by the instrument supplier, giving T-scores, defined as (BMDo - BMDm)/SD, where BMDo is the obtained BMD, BMDm is the mean value for 20-year-old Danish female controls, and SD is the standard deviation in the same reference population. For the purpose of this study, we defined low BMD as T-score < − 2.5 and moderately low BMD as T-score between − 2.5 < and <− 1.0, respectively.

### 2.3 Sub-cloning of OPG insert (TNFRSF11Bv2) and preparation of the pThOPG vector

The cDNA construct (TNFRSF11Bv2) prepared based on the sequence in the National Center for Biotechnology Information (NCBI) was from normal pigmented retinal epithelium human eye. The fragment was purchased in the pJ201 vector from DNA 2.0 Inc. (DNA 2.0 Inc. Menlo Park, CA) was cloned into the pTnT vector (Promega, Madison, WI). The pTnT™ vector was designed with multiple cloning sites to support highly efficient expression of cloned genes, was used for in vitro coupled transcription translation system in the presence of 35S-methionine. The cDNA construct (TNFRSF11Bv2) fragment was cut from the pJ201 vector (2 μg) (DNA2.0 Inc.) with EcoRI and NotI (FastDigestTM, Fermentas Sweden AB, Helsingborg, Sweden) using the FastDigest buffer 10 (Fermentas) using the restriction buffer, NEB2 10 × (New England Biolabs, Inc.). The linearized pTnT™vector and OPG construct were separated and analyzed by gel electrophoresis in 1% agarose and the two bands extracted using a gel purification kit (QIAquick Gel Extraction Kit, QIAGEN AB, Solna, Sweden) according to the manufacturer's instructions. Subcloning efficiency DH5α competent cells were used for transformation according to manufacturer's instructions (Invitrogen AB, Stockholm, Sweden). The pThOPG plasmid DNA was extracted using the QiaPrep Spin MiniPrep Kit (QIAGEN AB). The insert was sequenced by GATC Biotech AG (Konstanz, Germany) using the 5′-TTA CGC CAG CCCGGATCC-3′ and 5′-AAG GCT AGA GTA CTT AAT ACG A-3′ as the reverse and forward primers, respectively (DNA Technology A/S, Risskov, Denmark).

### 2.4 Coupled in vitro transcription–translation of the OPG plasmid (pThOPG)

The coupled in vitro transcription translation of the pThOPG vector were assembled in a single step one reaction mixture containing 2 μg of pThOPG (reaction concentration, 0,5 μg/μL), 50 μL TNT®rabbit reticulocyte lysate, 4 μL TNT®reaction buffer, 2 μL amino acid mixture without methionine, 2 μL RNasin® Ribonuclease inhibitor, 2 μL SP6 RNA Polymerase (all from Promega, Madison, WI), 4 μL [^35^S]-methionine (EasyTagTM, 1175 Ci/mmol Perkin Elmer, Boston) and nuclease-free water to a final volume of 100 μL. The reaction mixture was incubated for 90 min at 30 °C with shaking (300 rpm) in a ThermoMixer® comfort, Eppendorf, AG Hamburg, Germany). The translation product was subjected to a filtration Illustra™ NAP™-5 Columns (GE Healthcare, Buckinghamshire, UK) for removal of unincorporated label. The incorporation of [^35^S]-methionine radioactivity into the OPG peak fraction was counted in a β-counter (1450 MicroBeta®TriLux Microplate Scintillation and Luminescence Counter, (PerkinElmer Turku, Finland). and the concentration was determined (cpm/μL). The concentration of pThOPG was pre titrated for optimal protein expression for one reaction. The sum of the incorporated product, compared with total sum unincorporated product was calculated (cpm/μL). The incorporation yield of the [^35^S]-methionine radioactive label into the pTnT-rht OPG was calculated to an average 20%.

### 2.5 IgG-OPG autoantibody radiobinding assays (RBA)

Measurements of IgG-OPG autoantibodies were carried out by RBA as previously described for tissue transglutaminase autoantibodies with slight modifications (Kjelleras et al., 2011). In short, duplicate samples of 2.5 μL undiluted serum and a standard of rabbit anti human Osteoprotegerin (catalog no ab9986, Abcam, Cambridge, UK) diluted 1:10, 1:20, 1:40, 1:80, 1:160, 1:320, 1:640, 1:1280, 1:2560, and 1:5120 in antigen buffer (150 mmol/L NaCl, 20 mmol/L Tris, pH 7.4, 0.15% (v/v) Tween 20, 0.1% (w/v) BSA) and 60 μL labeled OPG-antigen at a final concentration of 425 ± 25 cpm/μL in antigen buffer were added in V-bottom 96-well plates (catalog no 442587, 96-well MicroWell™ PP plates, Nunc A/S, Roskilde, Denmark) and incubated over night at 4 °C on a plate shake (950 rpm) (Wallac Delfia® Plate Shake Perkin Elmer, Turku, Finland). A total of 50 μL of the serum and labeled OPG-antigen mixture was then transferred to a 96-well MultiScreen-DV Filter Plate (catalog nr MSDVN6B50, Merck Millipore S.A.S, Molsheim, France) (pre-coated with antigen buffer over night at 4 °C) and 50 μL 20% Protein A Sepharose 4B conjugated in antigen buffer (catalog nr 10-1090, Invitrogen, Thermo Fisher Scientific Inc, Carlsbad, CA) was added and incubated on a plate shake (950 rpm) for 90 min at 4 °C. The plate was then washed 8 times in antigen buffer using a microplate washer (ELx50TM Microplate Washer, BioTek Winooski, VT). 50 μL of scintillation cocktail OptiPhase Supermix (PerkinElmer Health Inc., Waltham, MA) was added. The antibody-antigen and Protein A Sepharose bound radioactivity was counted 1 min (cpm) in a β-counter.

### 2.6 Statistical analysis

**Fig. 1:**
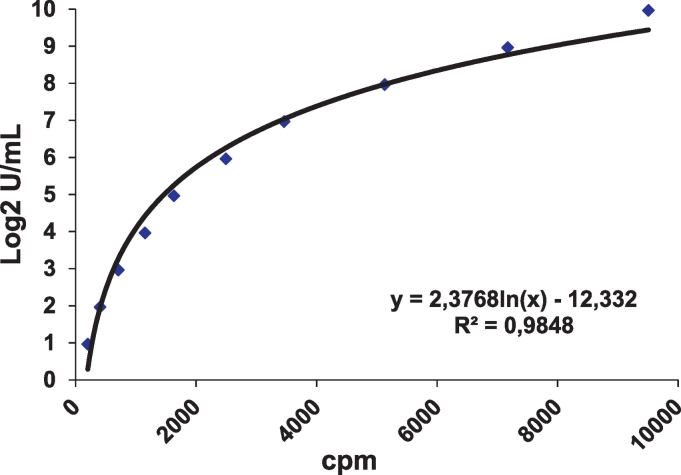
Nonlinear regression Log2 standard curve for IgG-OPG.

**Fig. 2:**
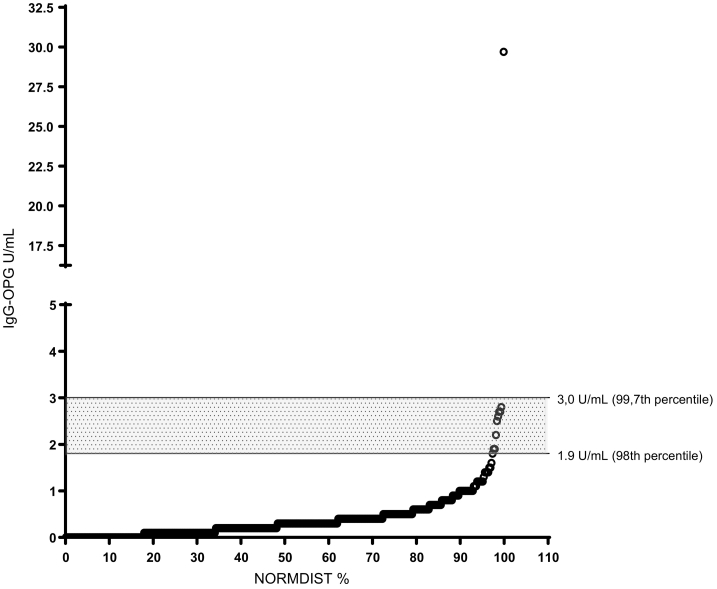
Quantile-quantile (QQ) plot for IgG-OPG in 398 healthy blood donors. The upper horizontal line denotes the cutoff level of positivity and lower line the cutoff level of borderline positivity, respectively.

The SPSS 18® statistical package (SPSS Inc. Chicago, IL) was used for statistical analysis. A *p* value 0.05 was considered as significant. Pearson Chi square test of independence (and Yates' correction for continuity value when applied) was used to assess differences in frequencies of autoantibody positivity. Levels of IgG-OPG were expressed as U/mL and calculated from standard curves. Background signal (antigen buffer) was subtracted from samples and standard curves (Fig. 1). Cut-off levels for a positive value were determined using quantile-quantile (QQ) plots to identify deviation from normality in the distribution of the 398 controls and set at > 3.0 U/mL representing the 99.7th percentile of 398 healthy blood donors (Fig. 2).

## 3 Results

### 3.1 Assay performance and prevalence of IgG-OPG

The sequencing of insert verified by GATC Biotech AG (Konstanz, Germany) showed no mutations in the sequence of the insert and the insert and vector were linked as expected. The assay showed the precision intra-assay CV was 8% for duplicates in IgG-OPG and reproducibility i.e. inter-assay CV was 7%, respectively. Forty-five of the 698 (6.6%) women were positive for IgG-OPG as compared with only 2 of the 398 (0.5%) controls (*p* < 0.0001).

### 3.2 Correlation between IgG-OPG and bone mineral density (BMD)

**Fig. 3:**
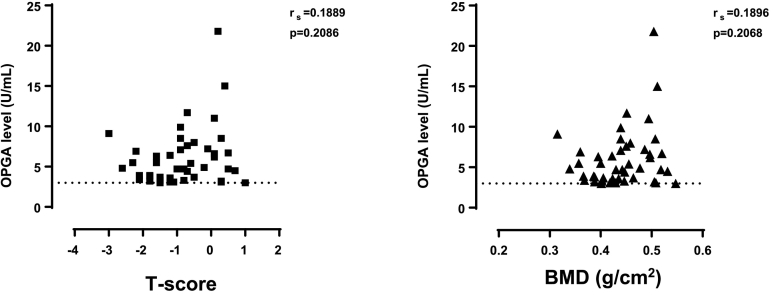
Correlations between levels of IgG-OPG and T-scores (left figure) respective bone mineral density (BMD) (right figure) in IgG-OPG positive individuals (*n* = 46). The dotted horizontal lines denote the cut-off levels of IgG-OPG-positivity.

The median BMD among the 698 women was 0.435 (range 0.176–0.652) g/cm2. Among those, 35 of 698 (5.0%) had low BMD with T-scores <− 2.5 and another 290 of 698 (41.5%) had moderately low BMD with T-scores between − 1- and <− 2.5. Among the 45 IgG-OPG positive women, 2 fulfilled the criteria of low BMD, 18 had moderately low BMD and the remaining 25 women had normal BMD. There was no difference in BMD and T-score between IgG-OPG positive and IgG-OPG negative individuals (Table 1). Neither was there any correlation between levels of IgG-OPG and BMD nor with T-scores in the IgG-OPG positive individuals (Fig. 3).

**Table 1:** Characteristics in IgG-OPG positive vs IgG-OPG negative individuals.

Variable^a^	IgG-OPG positive (*n* = 45)	IgG-OPG negative (*n* = 653)	*p*-Value
Age (years)	54.8 (50.8–60.6)	54.7 (50.4–60.6)	0.4956
IgG-OPG level (U/mL)	4.8 (3.0–21.8)	1.0 (0–2.9)	< 0.0001
Bone mass density (g/cm^2^)	0.439 (0.315–0.547)	0.435 (0.176–0.652)	0.3906
T-score	− 0.9 (− 3.0–1.0)	− 0.9 (− 3.8–2.8)	0.3442

a Median (range).

### 3.3 Predictive value of IgG-OPG for low and moderately low BMD

The diagnostic sensitivity for the OPG-RBA was 5.7% (i.e. 2 true IgG-OPG positive of 35 with low BMD) and the specificity was 92.9% (327 true IgG-OPG negative of 352 with normal BMD). The counterpart for moderately low BMD showed a diagnostic sensitivity of 5.8% (i.e. 18 true IgG-OPG positive of 310 with moderately low BMD) and specificity of 92.9%., respectively (i.e. 327 true IgG-OPG negative of 352 with normal BMD). The positive predictive value (PPV) for low BMD was 7.4% (i.e. 2 true IgG-OPG positive of 27 IgG-OPG positives) and for moderately low BMD 41.9% (18 true IgG-OPG positive of 43 IgG-OPG positives). The negative predictive value (NPV) for low BMD was 90.8% (i.e. 327 true IgG-OPG negative of 360 IgG-OPG negatives) and for moderately low BMD 52.8% (i.e. 327 true IgG-OPG negative of 619 IgG-OPG negatives).

## 4 Discussion

The present study set up a novel RBA for the measurement of serum OPG autoantibodies and evaluated their diagnostic performance to predict low BMD in middle-aged women previously assessed with DXA. The results clearly demonstrated that the diagnostic sensitivity and positive predictive value of the OPG autoantibody RBA was poor (< 8%) to identify individuals with a low or moderately low BMD. The conclusion from our results is therefore that OPG autoantibodies are not credible to distinguish individuals with a reduced BMD and that the diagnostic benefit of OPG autoantibodies is too inferior to be recommended as a screening marker for osteoporosis in the clinical setting.

The approach to define cut-off levels or deviation from normal for OPG autoantibodies may vary between laboratories, which method applied and the study population. In line with our results, a recent study detected OPG autoantibodies in 8.2% of patients with axial spondyloarthropathy or ankylosing spondylitis using an ELISA for OPG analysis (Hauser et al., 2017). In contrast to our findings, this previous study found that BMD total hip were significantly reduced in OPG autoantibody positive patients as compared to their controls (Hauser et al., 2017). In our study, we could not find any correlation between BMD or T-score and levels of OPG autoantibodies. A plausible explanation for the discordant results could be that we studied women recruited from the general population and not performed on patients at risk of osteoporosis.

It is also possible the disparities could be due to the fact that this study measured wrist BMD of the non-dominant wrist and not BMD of total hip. An analysis of spine and hip bone density on all subjects with reduced wrist BMD would indeed have been more meticulous. However, this was not practically or economically possible and is therefore a limitation of this study. Still, the method of wrist DXT technique through a supplementary dual-energy X-ray absorptiometry of hip BMD on nearly every fifth woman with a wrist T-score < − 2.5 SD was previously validated and showed a weak correlation between BMD of radius and hip (Lidfeldt et al., 2002). We therefore believe that performing DXA of the wrist was sufficiently credible to show accurate BMD also in this study, albeit a central DXA would probably identify the women with moderately low BMD more accurately.

It cannot be excluded that the use of different immunoprecipitation assays yield different results when OPG is kept in solution with a RBA as compared to when the antigen is immobilized in solid phase ELISA (Agardh et al., 2005). Since others have developed immunoprecipitation assays or ELISAs for the detection of OPG autoantibodies (Riches et al., 2009, Hauser et al., 2017), we may be criticized for not validating our RBA with previous established described assays and perform functional analysis. Real et al., reported that OPG autoantibodies showed a polyclonal response and the lack of association seen in the current study may therefore suggest that there may be a substantial proportion of non-functional OPG antibodies present (Real et al., 2015).

However, we adopted the same in-house RBA method that we have used in our laboratory for many years to assess autoantibodies against tissue transglutaminase, the screening marker for celiac disease (Agardh et al., 2003), as well as glutamic-acid decarboxylase 65 (GAD65) autoantibodies (Grubin et al., 1994) and zinc transporter 8 (ZnT8) to predict type 1 diabetes (Vaziri-Sani et al., 2010). For this study, we subcloned the original OPG cDNA into a high efficiency in vitro transcription translation plasmid (pTnT). Protein A Sepharose was used to bind autoantibodies against radiolabeled OPG. Protein A is produced by *Staphylococcous aureus* has the size of 42 kDa and contains five domains which are able to bind extra-cellular immunoglobulins to either the Fcγ fragment or to the VH3-region on the Fab fragment (Graille et al., 2000). Binding properties of protein A show strong affinity for IgG of all subtypes except for IgG3, but it also binds some of IgA and IgM (Ljungberg et al., 1993). The incorporation rate of ^35^S-methionine was consistently approximately 20% and the intra-assay variation for the IgG-OPG assay was 8%, quite similar to our previous immunoassays in use. It is therefore more likely that OPG autoantibodies are detected only in a subset of patients with autoimmune osteoporosis rather than the applied immunoassay used. Although other factors associated with osteoporosis were not accounted for is a limitation of the study, the association with BMD in an unbiased population of middle-age women selected from the general information has not previously been evaluated.

In conclusion, this population based study could not verify previous findings that OPG autoantibodies correlate with BMD in middle-age women. Still, OPG autoantibodies may be useful to identify a subset patient with autoimmune osteoporosis and may have functional inhibition of OPG as previously demonstrated in the original case report (Riches et al., 2009). Additional studies are therefore warranted to address the paradox of a relatively high prevalence of OPG autoantibodies in the general population without any clinical evidence of osteoporosis.
